# Integrating GBS-Derived SNP Markers with Phytochemical Profiling and Anti-Obesity Enzyme Inhibition in *Phyllanthus emblica*

**DOI:** 10.3390/molecules31111786

**Published:** 2026-05-22

**Authors:** Pimchanok Satapoomin, Thiplada Juntranon, Siriporn Sripinyowanich

**Affiliations:** 1 Department of Science and Bioinnovation, Faculty of Liberal Arts and Science, Kamphaeng Saen Campus, Kasetsart University, Nakhon Pathom 73140, Thailand; pimchanok.s@ku.th; 2 Plant Science Program, Department of Science and Bioinnovation, Faculty of Liberal Arts and Science, Kamphaeng Saen Campus, Kasetsart University, Nakhon Pathom 73140, Thailand; thiplada.ju@ku.th

**Keywords:** amla, anti-obesity, LC-MS/QTOF, genotyping-by-sequencing (GBS), SNP

## Abstract

*Phyllanthus emblica* L. is a nutraceutically important medicinal plant; however, the relationship between genetic variation and bioactive potential remains poorly understood. This study integrates genome-wide SNP analysis, phytochemical profiling, and functional bioassays to investigate cross-scale differentiation among fourteen cultivars. Genotyping-by-sequencing (GBS) identified 5644 high-quality SNPs from an initial dataset of 9018 SNPs, revealing moderate but structured genomic divergence (0.0275–0.0845). Phytochemical analysis of five commercial cultivars demonstrated significant variation (*p* < 0.05) in total phenolic content (6.58–15.53 mg GAE/gDW) and tannin content (284.52–333.81 mg TAE/gDW). Functional assays revealed strong anti-obesity potential, with crude extracts exhibiting superior α-glucosidase inhibition (up to 98.75%), while tannin-enriched extracts showed enhanced pancreatic lipase inhibition (up to 46.26%). Importantly, enzyme inhibition did not correlate directly with total phenolic or tannin content, indicating compound-specific bioactivity. LC-MS/QTOF analysis identified flavonoids (e.g., quercetin and kaempferol), phenolic acids, and other candidate metabolites potentially associated with enzyme inhibitory activity. These findings demonstrate a non-proportional association among genomic variation, metabolite composition, and functional bioactivity, suggesting that bioactivity may be influenced more strongly by compound-specific metabolite composition than by genome-wide similarity alone.

## 1. Introduction

*Phyllanthus emblica* L. (Indian gooseberry) is a medicinally significant plant widely utilized in traditional and contemporary therapeutic systems [1,2]. The fruit has been incorporated into herbal formulations and functional preparations due to its reported antioxidant capacity and its potential roles in metabolic regulation [3,4,5]. Extracts of *P. emblica* have been investigated for inhibitory activity against key digestive enzymes, including α-glucosidase and pancreatic lipase, which are relevant to carbohydrate and lipid metabolism [6,7,8]. These biological activities, particularly the anti-obesity potential of *P. emblica*, are largely attributed to its complex phytochemical composition, especially hydrolysable tannins and other phenolic constituents [9,10]. Phenolic compounds and flavonoids have been reported to interfere with carbohydrate and lipid digestion by inhibiting α-glucosidase and pancreatic lipase, thereby reducing nutrient absorption and contributing to metabolic regulation. This functional mechanism is consistent with current pharmacological strategies targeting obesity-associated metabolic pathways, including modulation of glucose metabolism, lipid digestion, appetite regulation, and energy homeostasis [11,12].

Despite its extensive medicinal use, the genetic architecture underlying cultivated *P. emblica* accessions remains insufficiently characterized [13]. Cultivars are typically distinguished by fruit morphology, agronomic performance, or geographical origin [14]; however, it remains unclear whether such classifications correspond to genome-wide differentiation or to localized genetic variability. The extent of accession-level genetic divergence and its relationship to variation in phytochemical composition and bioactivity have not been systematically examined across multiple biological scales [15].

Recent advances in genotyping-by-sequencing (GBS) and SNP-based population genomics have substantially improved the resolution of genetic diversity and population structure in medicinal plant species, enabling integrative investigation of genotype–phenotype relationships. However, interpretation of genetic differentiation is inherently resolution-dependent. Genome-wide approaches such as genotyping-by-sequencing (GBS) provide integrated assessments of overall genetic background by averaging variation across numerous loci [16]. While effective for capturing population-level structure, such approaches may attenuate signals arising from highly variable genomic regions. Conversely, targeted SNP markers focus on specific loci and may reveal pronounced divergence that does not necessarily represent genome-wide stratification [17]. As a result, relatedness inferred at different genomic resolutions may not be fully concordant, and the hierarchical structure of genetic variation within *P. emblica* cultivars remains incompletely resolved.

An additional unresolved issue concerns the relationship between genetic differentiation and medicinally relevant phenotypes. Although variation in phytochemical composition among *P. emblica* accessions has been reported [1,5,9,14], the propagation of genomic variability into metabolomic structure and functional enzyme inhibition has not been evaluated within an integrated framework. Genome-wide similarity does not inherently guarantee phytochemical similarity, and metabolic divergence does not necessarily translate proportionally into functional variation [18]. Without cross-scale analysis, the biological basis of variability in medicinal potential among cultivars remains only partially understood.

To address these gaps, the present study evaluated accession-level variation in *P. emblica* across hierarchical biological layers. Genome-wide GBS analysis was employed to establish background genetic relationships among cultivars, while targeted SNP analysis was used to assess fine-scale genomic differentiation. Representative cultivars spanning distinct phylogenetic positions were subsequently evaluated for phytochemical composition and enzyme inhibitory activity against α-glucosidase and pancreatic lipase. In addition, LC-MS/QTOF-based metabolite profiling was conducted on Emblica01, a representative accession, to characterize candidate bioactive metabolites potentially associated with the observed enzyme-inhibitory activities. Accordingly, this study aimed to investigate whether variation in genomic structure, phytochemical composition, and enzyme inhibitory activity exhibits coordinated or partially independent patterns across biological scales in *P. emblica*.

## 2. Results

### 2.1. Genome-Wide SNP Variation and Genetic Diversity Metrics

Genotyping-by-sequencing (GBS) analysis generated a total of 36,995,572 reads, of which 39.1% were successfully mapped to the reference genome (NCBI SRA ID SRP075209). The relatively moderate mapping rate is likely associated with the incomplete nature of the available reference genome, genomic divergence among accessions, and the reduced-representation characteristics of GBS libraries, which preferentially target highly variable restriction site-associated regions. Initial SNP calling identified 9018 SNPs, which were subsequently filtered to obtain 5644 high-confidence SNPs for downstream analysis (Table 1).

Across the fourteen *P. emblica* accessions, the total number of SNPs per accession ranged from 174,769 to 262,040, indicating substantial genomic variation within the population. Transition mutations were consistently more frequent than transversions, with ts/tv ratios ranging from 1.872 to 1.975 (Table 2), reflecting stable mutation patterns and supporting the overall quality of SNP detection. The heterozygosity rate (Het rate) varied from 1.111% to 1.756%, indicating moderate but uneven genetic diversity across accessions. Emblica09 exhibited the highest heterozygosity, whereas Emblica13 showed the lowest, suggesting differential levels of genomic variability among cultivars. These genomic metrics collectively indicate that *P. emblica* cultivars exhibit moderate but structured genetic diversity, providing a foundation for phylogenetic and downstream functional analyses.

### 2.2. Genotyping and SNP Analysis Reveal Structured Genomic Variation

**Figure 1:**
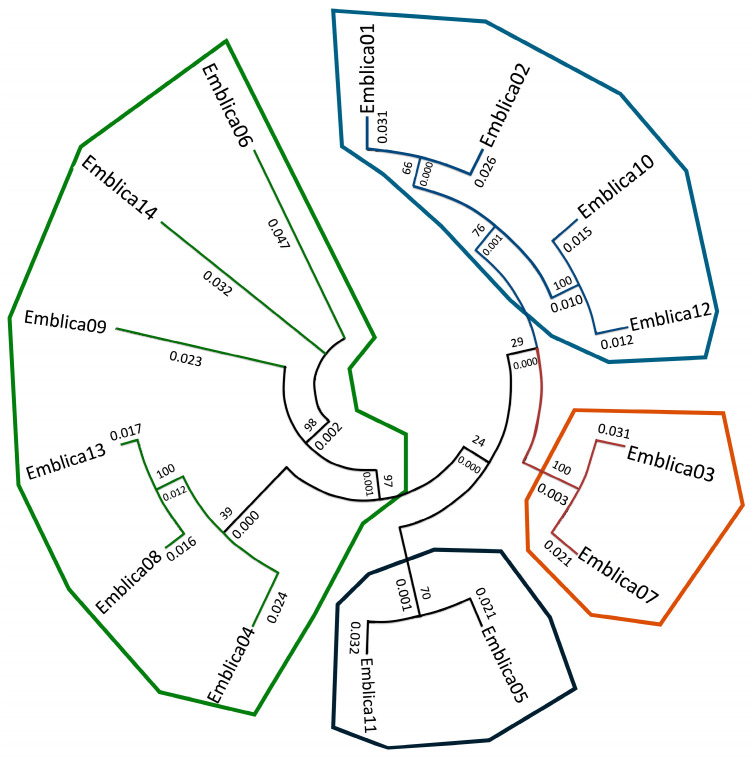
Phylogenetic relationships among fourteen *P. emblica* accessions were inferred using the neighbor-joining (NJ) method based on 5644 high-confidence SNPs derived from genotyping-by-sequencing (GBS) analysis. Bootstrap support values (%) based on 1000 replicates are indicated at the corresponding nodes. Branch lengths represent evolutionary distances calculated using the *p*-distance model. All ambiguous positions were removed using pairwise deletion, resulting in 32,585 positions in the final dataset. Phylogenetic analysis was performed using MEGA11 [19].

The filtered SNP dataset revealed moderate but structured genetic differentiation among the fourteen cultivars, with pairwise genetic distances ranging from 0.0275 to 0.0845. NJ clustering consistently resolved structured genomic relationships, indicating non-random divergence and the presence of defined genomic backgrounds across accessions. Phylogenetic analysis based on these SNP markers further resolved the cultivars into four distinct clusters (Figure 1), reflecting structured genomic organization across cultivated and wild germplasm. Cluster I comprised Yokmanee (Emblica01), India (Emblica02), Mae Luk Dok (Emblica10), and Pan Siam (Emblica12), with Mae Luk Dok and Pan Siam forming the closest pair (pairwise distance = 0.0275). Cluster II included Bunga (Emblica03) and To Lek (Emblica07), while Cluster III consisted of Phu Yai Sane (Emblica05) and Pan Si Thong (Emblica11). Cluster IV represented the most heterogeneous group, including Med Mayom (Emblica04), Pom Yak (Emblica06), To Yak (Emblica08), Bang Berd (Emblica09), Pan Khao (Emblica13), and the wild accession (Emblica14) (Figure 1). This phylogenetic structure indicates a hierarchical pattern of genomic organization, where closely related cultivar pairs are embedded within broader clusters. The inclusion of both cultivated and wild accessions within the same cluster suggests partial genomic continuity rather than complete separation driven by domestication.

To ensure that subsequent biochemical and functional analyses adequately represent the underlying genetic diversity, representative cultivars were selected from each phylogenetic cluster for downstream bioactivity evaluation. This phylogeny-guided sampling strategy enables systematic assessment of whether metabolomic composition and enzyme inhibitory activity correspond to genomic clustering or diverge across biological scales. By incorporating both closely related and genetically divergent accessions, the design provides a robust framework to evaluate the extent to which phylogenetic relationships predict functional traits.

Importantly, this approach highlights a key feature of *P. emblica* genomic architecture. While genome-wide analysis captures overall patterns of divergence, SNP-based distance and clustering reveal localized genomic coherence and heterogeneity, indicating that cultivar relationships are shaped by both global genomic background and locus-specific variation. Consequently, fine-scale genomic structuring offers enhanced resolution for interpreting accession-level relationships and their associated metabolomic and functional variation.

### 2.3. Nutraceutical Properties of Commercial Amla Varieties

**Figure 2:**
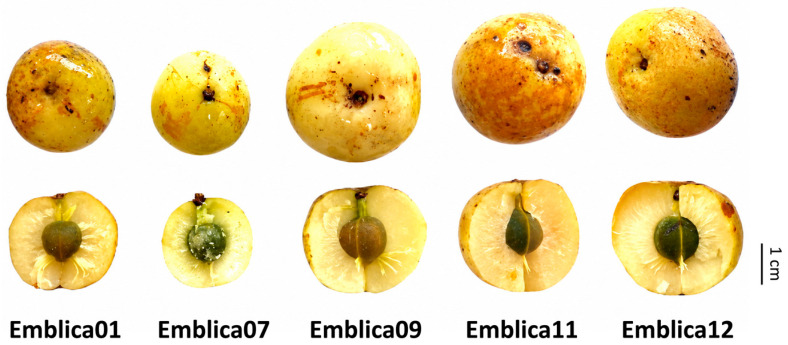
External (**top row**) and longitudinal cross-sectional (**bottom row**) views of fruits from five representative *P. emblica* cultivars: Yokmanee (Emblica01), To Lek (Emblica07), Bang Berd (Emblica09), Pan Si Thong (Emblica11), and Pan Siam (Emblica12).

Based on SNP-derived phylogenetic clustering, representative cultivars were selected from each major cluster to ensure coverage of the observed genomic diversity. Selection criteria included (i) representation of all phylogenetic clusters, (ii) inclusion of both tightly clustered and genetically divergent accessions, and (iii) availability of uniform plant material. Accordingly, five cultivars, including Yokmanee (Emblica01), To Lek (Emblica07), Bang Berd (Emblica09), Pan Si Thong (Emblica11), and Pan Siam (Emblica12), were chosen to represent distinct positions within the phylogenetic structure (Figure 2). This design enables cross-scale evaluation of whether metabolite composition and enzyme inhibitory activity follow genomic clustering or diverge across biological levels.

Bioactive compounds were extracted from five commercial varieties of amla using fruit pulp collected from a single cultivation site in Kanchanaburi province, Thailand. Two extraction methods were investigated: crude and tannin-enriched. Extraction yields ranged from 4.427% to 9.792% for crude ethanol extracts and from 11.370% to 15.064% for tannin-enriched extracts. Tannin-enriched extracts showed better tannin recovery than crude extracts, with the Bang Berd cultivar yielding the highest at 15.064%, as shown in Table 3. Although tannin-enriched extraction produced substantially greater overall extract yields than crude ethanolic extraction, the total tannin concentration within the extracts did not increase proportionally. This observation suggests that the aqueous alkaline extraction system recovered additional water-soluble constituents, including tannin-associated compounds, thereby increasing the total extract mass without necessarily increasing tannin concentration on a per-gram extract basis. Consequently, the higher extraction yield observed in tannin-enriched fractions likely reflects broader recovery of hydrophilic metabolites rather than selective enrichment of tannins alone.

The results showed that crude and tannin-enriched extracts from *P. emblica* contained notable levels of phenolic compounds and tannins, with significant differences influenced by cultivar, extraction method, and their interaction (Table 4). Among all combinations, the crude extract of Emblica01 exhibited the highest phenolic content (15.530 ± 0.248 mg GAE/g), while the tannin-enriched extract of Emblica11 also showed a high level (10.951 ± 0.053 mg GAE/g). These results are consistent with previous reports that *P. emblica* contains abundant polyphenols, particularly hydrolysable tannins. Tannin content was highest in crude extracts of Emblica09 and Emblica01, reaching over 333 mg/g. The standard product Capros^®^ exhibited the highest phenolic and tannin contents among all tested samples, confirming its commercial standardization. The results showed that *P. emblica* extracts contained substantial levels of phytochemicals, particularly phenolic compounds and tannins. These levels were significantly influenced by cultivar, extraction method, and their interaction (*p* < 0.001; Table 4). Crude extracts generally yielded higher total phenolic content (TPC), while tannin-enriched extracts exhibited stronger enzyme-inhibiting activity.

Regarding TPC, the highest value was found in the crude extract of Emblica01 (15.530 ± 0.248 mg GAE/g), followed by Emblica07 (12.651 ± 0.208 mg GAE/g). In contrast, the lowest TPC was observed in Emblica12 (6.583 ± 0.089 mg GAE/g). The control product Capros^®^ exhibited the highest phenolic content overall (81.713 ± 1.017 mg GAE/g). The substantially higher phenolic content in Capros^®^ reflects its standardized formulation, in contrast to the natural variability observed among cultivars. Total phenolic compounds are classified as polyphenolic bioactives with key roles in scavenging free hydroxyl radicals, thereby preventing lipid peroxidation and alleviating oxidative stress in biological systems. In this study, phenolic compounds were detected in both crude and tannin-enriched extracts of *P. emblica*, suggesting the presence of bioactive phytochemicals previously associated with antioxidant-related biological activities, including radical scavenging capacity and oxidative stress modulation [20,21,22]. Because direct antioxidant assays were not performed in the present study, antioxidant-related interpretations should be considered inferential and based primarily on the known biological associations of the detected phytochemical classes.

For total tannin content (TTC), the crude extracts of Emblica09 and Emblica01 recorded the highest concentrations (333.809 ± 4.410 and 333.095 ± 5.254 mg/g, respectively), while the lowest TTC was found in Emblica12 (284.524 ± 4.232 mg/g). The Capros^®^ control again showed the highest tannin content (343.095 ± 4.212 mg/g). Despite belonging to the same phylogenetic cluster (Cluster I), Emblica01 and Emblica12 showed significantly different metabolite profiles (*p* < 0.001; Table 4), indicating that phytochemical variation occurs independently of genome-wide genetic similarity.

### 2.4. Functional Enzyme Inhibition Reveals Selective Accession Sensitivity

Functional evaluation of representative *P. emblica* cultivars revealed distinct patterns of inhibitory activity against α-glucosidase and pancreatic lipase depending on extraction method and cultivar background (Table 4). Significant effects of cultivar, extraction method, and their interaction were observed for enzyme inhibitory activity and IC_50_ values (*p* < 0.001). Overall, crude extracts exhibited consistently strong α-glucosidase inhibition, with inhibition percentages ranging from 91.39% to 98.75%. Among the tested cultivars, the crude extract of Emblica12 showed the highest inhibitory activity (98.748 ± 0.251%), followed by Emblica07 (97.989 ± 0.691%), Emblica01 (96.568 ± 0.421%), and Emblica09 (96.501 ± 0.212%). These values exceeded or closely approached the inhibitory activity of the commercial reference product Capros^®^ (87.943 ± 0.333%). In contrast, tannin-enriched extracts generally exhibited lower α-glucosidase inhibition, revealing differential enzyme sensitivity between extraction systems. These results suggest that ethanol-soluble phytochemicals substantially contribute to α-glucosidase inhibitory activity in *P. emblica* extracts.

Dose–response analysis further demonstrated variation in inhibitory potency among cultivars and extraction types. Crude extracts generally exhibited lower IC_50_ values against α-glucosidase than tannin-enriched extracts, highlighting stronger inhibitory potency toward carbohydrate-digesting enzymes. Among the evaluated samples, the crude extract of Emblica12 exhibited the lowest IC_50_ value, consistent with the high inhibition observed in the screening assay. In contrast, tannin-enriched extracts showed comparatively higher IC_50_ values, indicating lower inhibitory potency against α-glucosidase despite containing substantial levels of tannin-associated metabolites. These findings indicate that α-glucosidase inhibition is not solely dependent on total phenolic or tannin abundance, but may instead reflect the contribution of specific flavonoids and ethanol-soluble phytochemicals enriched in crude extracts.

In contrast to α-glucosidase inhibition, pancreatic lipase inhibition exhibited greater variability among cultivars and extraction methods, supporting accession-dependent differentiation in lipid-digestion-related enzyme interactions. Crude extracts displayed relatively weak pancreatic lipase inhibition, with values ranging from 15.124% to 29.167%. Conversely, tannin-enriched extracts consistently demonstrated substantially stronger inhibitory activity. Among these, the tannin-enriched extract of Emblica12 exhibited the highest pancreatic lipase inhibition (46.263 ± 0.581%), followed by Emblica01 (41.514 ± 1.310%), Emblica07 (38.776 ± 2.442%), and Emblica09 (33.403 ± 3.799%). These activities were markedly higher than those of the commercial reference Capros^®^ (1.973 ± 0.752%).

Consistent with the inhibition percentages, tannin-enriched extracts generally exhibited lower IC_50_ values against pancreatic lipase than crude extracts, indicating stronger inhibitory potency toward lipid-digesting enzymes. Among the investigated accessions, the tannin-enriched extract of Emblica12 exhibited the lowest IC_50_ value, indicating the strongest pancreatic lipase inhibitory activity. The enhanced inhibition observed in tannin-enriched fractions may be associated with the selective enrichment of hydrolysable tannins and related phenolic derivatives, which have previously been implicated in lipase inhibition through protein-binding and enzyme conformational modification mechanisms. Nevertheless, IC_50_ values were not consistently proportional to total phenolic or tannin contents. For instance, although Capros^®^ exhibited the highest total phenolic and tannin levels, its inhibitory potency was not consistently superior to several *P. emblica* extracts. These observations indicate that enzyme inhibitory activity is influenced more strongly by qualitative metabolite composition and compound-specific interactions than by bulk phytochemical abundance alone.

Interestingly, cultivars belonging to the same phylogenetic cluster occasionally exhibited distinct inhibitory activities and IC_50_ profiles, whereas genetically divergent accessions sometimes demonstrated comparable enzyme-inhibitory potency. Such patterns suggest that functional bioactivity in *P. emblica* is not strictly determined by genome-wide similarity but is also shaped by downstream biochemical regulation and metabolite composition, which depends on extraction. Collectively, these findings support a partial genome–metabolite–function decoupling framework in which phylogenetic relatedness does not necessarily predict phytochemical functionality or enzyme inhibitory efficacy across cultivated *P. emblica* germplasm.

### 2.5. Identification of Candidate Bioactive Metabolites in Representative P. emblica Extracts

LC-MS/QTOF analysis was performed on crude and tannin-enriched extracts of Emblica01, selected as a representative accession exhibiting relatively high phytochemical content and strong enzyme inhibitory activity. The analysis revealed a chemically diverse metabolite composition comprising flavonoids, phenolic acids, amino acid derivatives, alkaloids, and organic acids (Table 5). Metabolites were putatively annotated based on accurate precursor ion mass (*m*/*z*), retention time, isotopic pattern, and spectral matching against publicly available metabolomics databases.

The ethanolic crude extract exhibited broad metabolite diversity and was particularly enriched in flavonoid-related compounds, including quercetin, quercetin-3′-O-glucoside, quercetin 7-rhamnoside, quercitrin, myricetin, kaempferol, and pinocembrin. Several metabolites displayed relatively high ion abundance under the analytical conditions employed, notably pipecolic acid (RT 1.66 min; area 9.203 × 10^6^), quercetin (RT 12.27 min; area 3.918 × 10^6^), trigonelline (RT 1.34 min; area 2.947 × 10^6^), cinnamic acid, and pyrrolidonecarboxylic acid. Nitrogen-containing metabolites, including arginine, proline, tryptophan, phenylalanine, and adenine derivatives, were also detected, indicating contributions from primary metabolic pathways.

In contrast, the tannin-enriched extract exhibited a comparatively more selective metabolite composition enriched in phenolic derivatives associated with hydrolysable tannin fractions. Major putatively annotated metabolites included ellagic acid, hyperin, cinnamic acid, quercetin 7-rhamnoside, and additional phenylpropanoid-related compounds. Pantothenic acid and pipecolic acid also exhibited relatively high ion abundance within the tannin-enriched fraction.

A semi-quantitative evaluation based on relative abundance (%) further demonstrated marked differences in metabolite distribution across extraction systems. In the crude extract, pipecolic acid, quercetin, trigonelline, cinnamic acid, and pyrrolidonecarboxylic acid represented major ion-abundant metabolites, whereas the tannin-enriched extract showed relative enrichment of pantothenic acid, pipecolic acid, cinnamic acid, indoleacrylic acid, hyperin, and phenolic derivatives associated with hydrolysable tannin-related compounds (Table 5). These findings indicate that the extraction strategy substantially influences relative metabolite composition, which may contribute to the observed variation in enzyme inhibitory activity and IC_50_ values.

Several metabolites detected in both extraction systems have previously been associated with α-glucosidase and pancreatic lipase inhibitory activity, particularly flavonoids and hydrolysable tannin-related compounds. The differential enrichment of these metabolites between crude and tannin-enriched extracts may therefore contribute to the observed variation in inhibitory potency. However, because LC-MS/QTOF profiling was performed only for the representative accession Emblica01, direct metabolomic comparison among cultivars was beyond the scope of the present study. Consequently, the identified metabolites should be interpreted as candidate bioactive constituents potentially associated with the observed enzyme-inhibitory activities rather than as definitive cultivar-specific metabolic determinants.

Comparative profiling revealed clear differences between extract types. The crude extract exhibited greater chemical diversity, particularly in flavonoid composition, whereas the tannin-enriched extract showed selective enrichment of phenolic derivatives, including phenolic and hydrolysable tannin-associated metabolites. Additional aromatic and phenylpropanoid-related compounds, such as coumarin, cinnamic acid, and anisaldehyde, were detected, highlighting metabolites linked to phenolic biosynthetic pathways. LC-MS/QTOF analysis demonstrates that the crude and tannin-enriched extracts differ substantially in metabolite composition, with the former characterized by broad chemical diversity and the latter by selective enrichment of phenolic and tannin-related compounds. These compositional differences correspond to the observed variation in enzyme inhibitory activity between extract types.

### 2.6. Relationships Between Genomic Structure, Phytochemical Composition, and Functional Bioactivity

To evaluate whether genomic relationships were associated with phytochemical variation and enzyme inhibitory activity, phylogenetic clustering was compared with phytochemical characteristics and functional bioactivity across *P. emblica* cultivars. Phylogenetic analysis resolved the accessions into four major clusters with clear subgrouping patterns, supporting structured genomic differentiation among cultivated and wild germplasm. However, genomic similarity did not consistently correlate with variation in phytochemical content or enzyme-inhibitory activity among cultivars.

For example, within Cluster I, Mae Luk Dok (Emblica10) and Pan Siam (Emblica12) exhibited the closest genetic relationship (distance = 0.0275), reflecting strong genome-wide similarity. Despite this, these cultivars exhibited distinct phytochemical profiles and enzyme-inhibition patterns, particularly in pancreatic lipase inhibition. Similarly, cultivars within Cluster IV, which exhibited comparatively greater genetic heterogeneity, did not consistently show proportionally greater variation in functional bioactivity. These observations suggest that biochemical and functional traits may not scale directly with genome-wide genetic divergence.

At the phytochemical level, significant variation in total phenolic and tannin contents was observed among cultivars and extraction methods. However, these variations did not consistently align with phylogenetic clustering patterns. Cultivars belonging to different genetic clusters occasionally exhibited comparable phenolic and tannin levels, whereas genetically related cultivars sometimes displayed distinct phytochemical characteristics. These findings suggest that phytochemical variation in *P. emblica* may not be fully explained by genome-wide similarity alone and could additionally be influenced by regulatory, biochemical, or environmental factors.

Functional assays further supported partial independence between genomic similarity and enzyme inhibitory activity. α-Glucosidase inhibition remained consistently high across most cultivars, particularly in crude extracts, suggesting relatively conserved inhibitory capacity toward carbohydrate-digesting enzymes. In contrast, pancreatic lipase inhibition exhibited substantially greater variability, especially in tannin-enriched extracts. Dose–response analysis and IC_50_ determination further demonstrated that inhibitory potency was not consistently proportional to total phenolic or tannin content. For example, several extracts with comparatively lower phytochemical abundance exhibited stronger inhibitory potency than samples with higher total phenolic or tannin levels. These observations suggest that enzyme inhibitory activity may be influenced more strongly by qualitative differences in bioactive constituents and compound-specific interactions than by overall phytochemical abundance alone.

Importantly, LC-MS/QTOF profiling was performed only on the representative accession Emblica01; therefore, direct metabolomic comparison among cultivars was beyond the scope of the present study. Consequently, the present findings should be interpreted as evidence of partial independence among genomic structure, phytochemical characteristics, and functional bioactivity rather than as definitive metabolomic evidence of cultivar-specific enzyme-inhibitory mechanisms. Nevertheless, the results provide an integrative framework for future comparative studies investigating genome–metabolite–function relationships in *P. emblica*.

## 3. Discussion

This study integrates GBS-derived SNP analysis, phytochemical characterization, representative LC-MS/QTOF metabolite profiling, and enzyme inhibitory assays to investigate hierarchical relationships among genomic structure, phytochemical variation, and functional bioactivity in *P. emblica*. The results demonstrate that although SNP-based phylogenetic analysis effectively resolved genetic structure among cultivars, phylogenetic proximity did not consistently correspond to metabolite composition or enzyme inhibitory activity. GBS analysis generated 5644 high-confidence SNPs, revealing moderate but structured genomic differentiation (0.0275–0.0845), consistent with previous reports demonstrating that SNP markers effectively resolve intra-species diversity in medicinal plants [23,24]. The observed heterozygosity levels (1.111–1.756%) indicate moderate genetic diversity within the cultivated *P. emblica* germplasm. Such heterozygosity is commonly observed in perennial fruit species and medicinal plant germplasm, where historical outcrossing, partial domestication, and mixed propagation practices contribute to the maintenance of genomic variation across cultivated accessions [25,26,27]. Moderate mapping efficiency is commonly observed in GBS analyses of non-model plant species because reduced-representation sequencing preferentially captures highly polymorphic genomic regions that may exhibit incomplete correspondence with available reference assemblies [28,29]. Despite the relatively moderate mapping rate observed in this study, stringent SNP filtering retained high-confidence variants suitable for phylogenetic reconstruction and diversity analysis [30]. However, genetically related accessions occasionally exhibited distinct biochemical and functional characteristics. For example, Emblica10 and Emblica12, which represented the closest genetic pair, displayed different phytochemical compositions and enzyme inhibition patterns, indicating that genome-wide similarity alone is insufficient to predict functional traits.

Phytochemical analysis further supports this partial disconnect between genomic structure and biochemical composition. Significant variation in total phenolic and tannin contents was observed among cultivars and extraction systems (*p* < 0.001), yet these differences did not consistently align with phylogenetic clustering patterns. Similar observations have been reported in medicinal plants, where secondary metabolite accumulation is influenced not only by genomic background but also by transcriptional regulation, environmental conditions, developmental stage, and pathway-specific metabolic control [15,31,32]. The higher extraction yield observed in tannin-enriched fractions did not necessarily correspond to greater tannin concentration. Under aqueous alkaline extraction conditions, broader recovery of polar and water-soluble constituents, including soluble carbohydrates, amino acids, organic acids, and other phenolic-associated metabolites, likely contributed to increased extract mass [33,34]. Consequently, the extraction yield reflects total recovered soluble material rather than selective tannin concentration alone. The detection of non-tannin metabolites within tannin-enriched fractions further indicates partial extraction selectivity, producing a phenolic-enriched metabolite mixture rather than a chemically purified tannin isolate [34,35].

Functional assays revealed extraction-dependent differences in enzyme inhibitory activity. α-Glucosidase inhibition remained consistently high across cultivars (>91%), suggesting a relatively conserved functional trait associated with broadly distributed phenolic compounds. Flavonoids such as quercetin and kaempferol have previously been reported to inhibit α-glucosidase through competitive and non-competitive interactions with catalytic residues [36]. In contrast, pancreatic lipase inhibition exhibited substantially greater variability, particularly in tannin-enriched extracts, indicating more pronounced compound-specific effects. Hydrolysable tannins and related phenolic compounds are known to inhibit pancreatic lipase through protein binding and conformational modification mechanisms [37]. Because a pharmaceutical lipase inhibitor was not included as a positive control, the present pancreatic lipase assay should be interpreted as a comparative functional evaluation rather than a direct pharmacological efficacy assessment. Furthermore, dose–response analysis and IC_50_ determination demonstrated that inhibitory potency was not consistently proportional to total phenolic or tannin abundance. These observations collectively suggest that qualitative metabolite composition and interactions among specific bioactive constituents contribute more substantially to enzyme inhibitory potency than bulk phytochemical abundance alone. Because the present study was limited to in vitro enzyme inhibition assays, the observed activities should be interpreted as comparative functional interactions rather than direct evidence of anti-obesity efficacy. Although IC_50_ values were determined to comparatively evaluate inhibitory potency, comprehensive enzyme kinetic characterization, including inhibition mode and binding dynamics, was beyond the scope of the present study.

Representative LC-MS/QTOF profiling of Emblica01 provided additional molecular insight into these extraction-dependent functional differences. The crude extract exhibited broad metabolite diversity enriched in flavonoid-related metabolites, including quercetin derivatives, quercitrin, kaempferol, and pinocembrin. In contrast, the tannin-enriched extract exhibited relatively greater enrichment of hydrolysable tannin-associated metabolites, including ellagic acid and hyperin. Several of these metabolites have previously been associated with α-glucosidase and pancreatic lipase inhibitory activity [15,36]. Flavonoids such as quercetin and kaempferol may inhibit α-glucosidase through interactions with catalytic residues, whereas hydrolysable tannins and related phenolic compounds may interfere with pancreatic lipase activity through protein-binding mechanisms and conformational modification of the enzyme [18,38]. The observed differences in relative metabolite abundance between crude and tannin-enriched extracts further support the interpretation that extraction-dependent metabolite redistribution, rather than total phenolic concentration alone, contributes substantially to variation in enzyme inhibitory activity. Specifically, selective enrichment of flavonoid-related metabolites in crude extracts and hydrolysable tannin-associated compounds in tannin-enriched fractions may differentially influence α-glucosidase and pancreatic lipase inhibition, respectively.

Although genome-wide SNP analysis revealed structured phylogenetic differentiation among the 14 accessions, comprehensive metabolomic profiling was not performed across the entire cultivar set. Consequently, the present study could not directly establish accession-specific genome–metabolite relationships. Instead, LC-MS/QTOF analysis was employed to provide representative compositional characterization of extraction systems associated with functional bioactivity. Because LC-MS/QTOF profiling was conducted only for Emblica01 extracts, the present study does not comprehensively resolve cultivar-specific metabolomic differentiation across the entire germplasm population. Future studies involving untargeted metabolomic profiling across all representative accessions will be necessary to directly evaluate genome–metabolite relationships in *P. emblica*. The discrepancy between phytochemical abundance and functional activity was further illustrated by the commercial reference product Capros^®^, which exhibited the highest total phenolic and tannin contents but did not consistently demonstrate superior enzyme inhibitory potency. This observation is consistent with previous studies indicating that total phenolic content alone is not always predictive of biological activity [39], thereby reinforcing the importance of compound-specific metabolite composition. These findings collectively suggest partial non-proportional relationships among genomic structure, phytochemical composition, and functional bioactivity in *P. emblica*. Similar relationship patterns have been reported in metabolomics studies, in which metabolite composition does not strictly follow phylogenetic relationships [39].

Nevertheless, several limitations should be acknowledged. LC-MS/QTOF profiling was conducted only for the representative accession Emblica01; therefore, comparative metabolomic differentiation across all cultivars could not be comprehensively resolved. In addition, metabolite annotation remained putative and requires further validation using authentic reference standards and MS/MS fragmentation analyses. Future studies integrating comparative metabolomics, transcriptomics, and pathway-level analyses across multiple accessions will be necessary to more comprehensively resolve genome–metabolite–function relationships in *P. emblica*.

## 4. Materials and Methods

### 4.1. Plant Materials and Sample Collection

A total of fourteen cultivars of *Phyllanthus emblica* grown in Thailand were used for genetic diversity analysis. Young leaves were collected from healthy plants, rapidly frozen in liquid nitrogen, and stored at −80 °C until use. Samples were powdered in liquid nitrogen using a mortar and pestle, and genomic DNA was extracted using the CTAB method [40]. The quality and concentration of the extracted genomic DNA were assessed using a Nanodrop (Thermo Fisher Scientific, Waltham, MA, USA) and 1% agarose gel electrophoresis. For nutraceutical evaluation, five commercial amla varieties, namely Emblica01, Emblica07, Emblica09, Emblica11, and Emblica12 (Figure 1), were selected based on observed genomic diversity. The fruits were carefully washed to remove any surface contaminants. The fruit pulp was then sliced into small pieces, approximately 0.5 cm thick. The sliced fruit pieces were freeze-dried at −40 °C and subsequently stored at −20 °C throughout the experimental period.

### 4.2. DNA Extraction and Genotyping-by-Sequencing (GBS)

DNA quality and concentration were assessed before library preparation. Genotyping-by-sequencing (GBS) libraries were constructed as described by Peterson et al. [24] following a reduced-representation sequencing strategy. Briefly, 100 ng of genomic DNA were enzymatically digested using *EcoRI* and *MspI* (NEB, Ispwich, MA, USA) and ligated to *EcoRI* and *MspI* adaptors. Each sample was assigned a unique barcode and PCR amplicons of all samples were equimolarly pooled together into two libraries. PCR amplicons were cleaned using AMPure XP beads (Beckman Coulter, Brea, CA, USA) with equal ratios. The quality of amplicons was measured using a 5200-fragment analyzer (Agilent Technologies, Santa Clara, CA, USA). Libraries were sequenced on the Illumina Hi-Seq 2500 platform (Illumina Inc., San Diego, CA, USA), yielding paired-end reads. Single-nucleotide polymorphism (SNP) calling was performed using the reference-based GBS pipeline [41] as described by Tesfamiceal et al. [42]. Before SNP calling, sequence reads were filtered for those that matched barcodes, had a minimum kmer count (<10), and had a kmer length (<20). To deduce the genomic position of read tags, sequence reads were mapped to the reference genome (NCBI SRA ID SRP075209).

### 4.3. SNP Calling, Filtering, and Genetic Analysis

SNP calling was conducted using a standardized bioinformatics pipeline. SNPs with low call rates, excessive missing data, or low minor allele frequency were excluded to ensure data reliability. A total of 5644 high-quality SNPs were obtained and used for genetic diversity analysis.

Phylogenetic relationships among amla varieties were inferred using the neighbor-joining method in MEGA 11 software [19]. Genetic distance matrices were calculated, and phylogenetic trees were constructed to visualize genetic relationships among accessions.

### 4.4. Preparation of Amla Extracts

Fresh amla fruits from the five selected commercial varieties were processed for nutraceutical analysis. Ethanol extraction was performed to obtain crude extracts rich in bioactive compounds. In addition, tannin-enriched extracts were prepared using appropriate extraction procedures to evaluate enzyme inhibitory activities. Freeze-dried amla (50 g) was weighed and extracted with 200 mL of 70% (*v*/*v*) ethanol in a 500 mL Erlenmeyer flask. The flask was tightly sealed and incubated in a shaking water bath at 50 °C for 24 h. The resulting mixture was filtered through Whatman No. 1 filter paper to remove residue. Then, the filtrate was evaporated to dryness at 65 °C for 2–4 h.

The tannin-enriched extraction procedure was performed to selectively fractionate tannin-associated phenolic compounds and reduce the broader metabolite complexity present in crude ethanolic extracts, thereby enabling comparative evaluation of composition-dependent bioactivity. Another 50 g portion of the freeze-dried fruit powder was finely ground and mixed with 200 mL of distilled water. Sodium hydroxide (NaOH, 2 g) was added to facilitate the solubilization of tannin. The mixture was incubated in a shaking water bath at 80 °C for 1 h. After incubation, the mixture was filtered through Whatman No. 1 filter paper (Cytiva, Marlborough, MA, USA) to obtain a clear extract, which was then concentrated to dryness using a rotary evaporator. The dried extract was weighed to determine the extraction yield (Equation (1)), then redissolved in distilled water to a final concentration of 100 mg/mL and stored at −20 °C until further use.

(1) $$Extraction\;yield\;(\%\;w/w)=\frac{Weight\;of\;dried\;extract\;(g)}{Initial\;dry\;sample\;weight\;(g)}\times 100$$

### 4.5. LC-MS/QTOF Analysis and Putative Metabolite Annotation

LC-MS/QTOF analysis was performed using three biological replicates to characterize the metabolite composition of crude ethanolic and tannin-enriched extracts of *P. emblica* (Emblica01), selected as a representative accession exhibiting relatively strong enzyme inhibitory activity and high phytochemical content. Metabolite profiling was conducted using an Agilent 1100 series ultra-high-performance liquid chromatography (UHPLC) system (Agilent Technologies, Santa Clara, CA, USA) coupled with a quadrupole time-of-flight mass spectrometer (QTOF-MS; API 4000) (Applied Biosystems Inc., Framingham, MA, USA) operated in positive electrospray ionization mode. Chromatographic separation was carried out using Zorbax SB-C18 (Agilent Technologies, Santa Clara, CA, USA) and Atlantis-d C18 (Waters Corporation, Milford, MA, USA) columns under optimized gradient elution conditions. The mobile phase consisted of H_2_O:MeOH (3:1, *v*/*v*) and acetonitrile, delivered at a flow rate of 0.6 mL/min with a total runtime of 15 min. Mass spectrometric conditions included an ion spray voltage of 5200 V, source temperature of 450 °C, nitrogen collision gas at 5 psig, curtain gas at 13 psig, nebulizer gas (GAS-1) at 52 psig, heater gas (GAS-2) at 58 psig, and entrance potential (EP) of 10 V. Raw LC-MS/QTOF data were processed for peak detection, deconvolution, alignment, and feature extraction prior to metabolite annotation. Putative metabolite annotation was performed based on accurate precursor ion mass (*m*/*z*), retention time (RT), isotopic distribution, adduct formation patterns, and MS/MS spectral matching against publicly available metabolomics databases, including METLIN, HMDB, and MassBank.

Metabolites were assigned as Level 2 putative annotations according to the Metabolomics Standards Initiative (MSI) guidelines because authentic reference standards were not used for definitive compound confirmation. Relative metabolite abundance (%) was calculated from normalized chromatographic peak area relative to the total detected ion peak area within each extract and was used for comparative semi-quantitative metabolite profiling under identical analytical conditions. Representative total ion chromatograms (TICs), extracted ion chromatograms (EICs), and selected MS/MS fragmentation spectra information of major metabolites are provided in Supplementary Materials. The present LC-MS/QTOF analysis was intended for comparative metabolite profiling rather than absolute quantitative metabolomics.

### 4.6. Determination of Total Phenolic Content

The total phenolic content of all crude ethanolic and tannin-enriched extracts was determined using the Folin–Ciocalteu assay [43] with slight modifications. A volume of 0.2 mL of each extract was mixed with 2.5 mL of distilled water. Then, 0.2 mL of 10% (*v*/*v*) Folin–Ciocalteu reagent was added, followed by the addition of 2.0 mL of 7.5% (*w*/*v*) sodium carbonate solution. The resulting mixture was incubated in the dark at room temperature for 90 min. The absorbance was measured at 765 nm using a UV-Vis spectrophotometer. Absolute ethanol was used as the blank, and gallic acid at concentrations of 0, 50, 100, and 150 mg/L was used to construct the calibration curve. The total phenolic content was expressed as milligrams of gallic acid equivalent per gram of extract (mg GAE/g extract).

### 4.7. Determination of Hydrolysable Tannin Content

The hydrolysable tannin content of crude ethanolic extracts and tannin-enriched extracts from five cultivars was determined using a modified method [44]. Briefly, each extract was prepared at a concentration of 10 mg/mL. An aliquot of 0.2 mL of the extract was mixed with 2.5 mL of distilled water, then 0.2 mL of 10% Folin–Ciocalteu reagent was added. After mixing, 2.0 mL of 7.5% sodium carbonate solution was added, and the reaction mixture was incubated in the dark at room temperature for 90 min. The absorbance was measured at 765 nm using a UV-Vis spectrophotometer.

### 4.8. Anti-Obesity Enzyme Inhibition Assay of Amla Extracts

#### 4.8.1. Inhibition of α-Glucosidase Enzyme

The α-glucosidase inhibitory activity of the extracts was assessed using a *p*-nitrophenol colorimetric assay, modified from the method described by [39]. Capros^®^ (Natreon, Inc., New Brunswick, NJ, USA) was used as the positive control. The assay is based on the hydrolysis of p-nitrophenyl-α-D-glucopyranoside (PNP-G) by α-glucosidase, which releases glucose and p-nitrophenol, producing a yellow color detectable at 405 nm. In the assay, 20 µL of extract (10 mg/mL), 100 µL of 50 mM sodium phosphate buffer (pH 6.8), and 20 µL of α-glucosidase solution (1.0 U/mL in phosphate buffer, pH 6.8) were mixed in test tubes and incubated at room temperature for 10 min. After pre-incubation, 20 µL of 2.0 mM PNP-G was added to initiate the reaction. The mixture was shaken gently and incubated at room temperature for an additional 5 min. Finally, 40 µL of 1.0 mM sodium carbonate (Na_2_CO_3_) solution was added to stop the reaction, and the absorbance was measured at 405 nm using a UV-Vis spectrophotometer (Shimadzu UV-1800, Shimadzu Corporation, Kyoto, Japan).

#### 4.8.2. Inhibition of Pancreatic Lipase Enzyme

The inhibitory activity of the extracts against pancreatic lipase was assessed using a previously described method [45], with minor modifications. The assay was based on a colorimetric method that measures the release of p-nitrophenol from *p*-nitrophenyl laurate (Sigma-Aldrich, St. Louis, MO, USA), the substrate. Upon hydrolysis by pancreatic lipase type II (Sigma-Aldrich, St. Louis, MO, USA), the substrate yields p-nitrophenol and lauric acid as products. The formation of p-nitrophenol was monitored by measuring the absorbance at 405 nm using a UV-Vis spectrophotometer. Capros^®^ (Natreon, Inc., New Brunswick, NJ, USA) was used as the positive control. The percentage inhibition of enzymes was calculated using the following Equation (2).

(2) $$Enzyme\;activity\;inhibition\;(\%)=\left[\frac{\left(A-B\right)}{A}\right]\times 100$$

where A is the absorbance of the reaction mixture without the test sample (control), and B is the absorbance of the reaction mixture containing the test sample (extract).

#### 4.8.3. Dose–Response Analysis and IC_50_ Determination

To quantitatively evaluate enzyme inhibitory potency, dose–response assays were performed to assess inhibition of α-glucosidase and pancreatic lipase using selected *P. emblica* extracts. Extract stock solutions were serially diluted to obtain final concentrations ranging from 0.5 to 10 mg/mL Each concentration was evaluated under the same assay conditions described above for α-glucosidase and pancreatic lipase inhibition. For each extract concentration, enzyme inhibition (%) was calculated relative to the control reaction using Equation (2). Dose–response curves were constructed by plotting inhibition percentage against extract concentration. IC_50_ values, defined as the concentration required to inhibit 50% of enzyme activity, were estimated using nonlinear regression analysis based on a four-parameter logistic model. All assays were conducted in triplicate, and IC_50_ values were expressed as mean ± standard deviation.

### 4.9. Statistical Analysis

All experiments were performed in triplicate, and the results are presented as mean ± standard deviation (SD). Statistical analyses were conducted using IBM SPSS Statistics software version 20.0 (IBM Corp., Armonk, NY, USA). Prior to statistical analysis, the assumptions required for parametric testing were evaluated. Data normality was assessed using the Shapiro–Wilk test, Kolmogorov–Smirnov test, and histogram distribution analysis, while Levene’s test was used to verify the homogeneity of variances for the experimental data sets.

The effects of cultivar (V), extraction method (E), and their interaction (V × E) on total phenolic content (TPC), total tannin content (TTC), α-glucosidase inhibitory activity, pancreatic lipase inhibitory activity, and IC_50_ values were analyzed using two-way analysis of variance (ANOVA) under a completely randomized design (CRD). When significant differences were detected, mean comparisons were performed using Duncan’s multiple range test (DMRT) at a significance level of *p* < 0.05. In cases where the homogeneity of variance assumption was violated, Welch ANOVA and Games–Howell post hoc tests were used as robust alternatives for unequal variances. Statistical significance levels for main effects and interactions are reported as *, **, and *** corresponding to *p* < 0.05, *p* < 0.01, and *p* < 0.001, respectively, while “ns” indicates non-significant differences. The coefficient of variation (CV, %) was calculated to assess experimental precision. Calibration curve slopes and coefficients of determination (R^2^) were calculated using Microsoft Excel 2019 (Microsoft Corp., Redmond, WA, USA).

## 5. Conclusions

This study integrates genome-wide SNP genotyping, phytochemical characterization, representative LC-MS/QTOF metabolite profiling, and functional enzyme inhibition assays to investigate biological variation among *Phyllanthus emblica* accessions across genomic, biochemical, and functional scales. GBS-derived SNP analysis generated 5644 high-confidence markers and resolved the evaluated germplasm into four phylogenetic clusters, revealing moderate but structured genomic differentiation among cultivated and wild accessions. Guided by this SNP-based framework, representative cultivars spanning distinct phylogenetic positions were subsequently selected for phytochemical and functional evaluation, which revealed substantial variation in extract yield, total phenolic and tannin contents, and enzyme inhibitory activity across cultivars and extraction systems.

Functional assays demonstrated pronounced extraction-dependent differences in bioactivity. Crude extracts consistently exhibited strong α-glucosidase inhibitory activity, whereas tannin-enriched extracts generally showed greater pancreatic lipase inhibitory potency. Dose–response analysis and IC_50_ determination further demonstrated that inhibitory potency was not consistently proportional to total phenolic or tannin abundance, indicating that bulk phytochemical content alone may not sufficiently explain functional activity. Representative LC-MS/QTOF profiling of Emblica01 identified multiple candidate bioactive metabolites, including flavonoid-related and hydrolysable tannin-associated compounds potentially involved in enzyme inhibition. The differential enrichment of these metabolites between crude and tannin-enriched extracts may contribute to the observed variation in inhibitory potency between extraction systems.

Importantly, phylogenetic proximity did not consistently correlate with phytochemical characteristics or functional bioactivity across cultivars. Genetically related accessions occasionally exhibited distinct biochemical and enzyme inhibitory profiles, whereas genetically divergent cultivars sometimes displayed comparable functional activity. Collectively, these findings suggest that bioactivity in *P. emblica* may be influenced not only by genome-wide genetic background and overall phytochemical abundance but also by qualitative metabolite composition and compound-specific biochemical interactions.

Several limitations should nevertheless be acknowledged. LC-MS/QTOF profiling was conducted only for the representative accession Emblica01; therefore, direct comparative metabolomic analysis among all cultivars was beyond the scope of the present study. In addition, metabolite annotation remained putative and requires further validation using authentic standards and MS/MS fragmentation analyses. Accordingly, the present findings should be interpreted as evidence supporting partial independence between genomic structure, phytochemical variation, and functional bioactivity rather than definitive metabolomic determination of cultivar-specific enzyme inhibitory mechanisms.

Overall, this work establishes an integrated genomic-phytochemical-functional framework for evaluating *P. emblica* germplasm and highlights the value of combining SNP-based genotyping with phytochemical and bioactivity analyses for nutraceutical and functional food applications. Future studies integrating comparative metabolomics, transcriptomics, quantitative pathway analyses, and authentic-standard-based metabolite validation across multiple cultivars will further clarify the regulatory mechanisms linking genomic variation, metabolite accumulation, and health-related bioactivity in *P. emblica*.

## Figures and Tables

**Table 1:** Summary of GBS data in 14 amla accessions.

Item	Total
Number of reads	36,995,572
Average length per read (bp)	100
Length of total read (bp)	5,363,247,188
Number of raw SNPs	9018
Number of filtered SNPs	5644

**Table 2:** Summary of SNP variation and genetic diversity metrics among fourteen *P. emblica* accessions. The table presents the number of transition (ts) and transversion (tv) mutations, the ts/tv ratio, heterozygosity rate (Het rate, %), and the total number of SNPs identified in each accession based on GBS-derived high-confidence variants. The ts/tv ratio serves as an indicator of SNP-calling quality and mutation-pattern consistency, while the heterozygosity rate reflects within-accession genetic variation.

Accession	Cultivars	ts	tv	ts/tv	Het Rate (%)	Total
Emblica01	Yokmanee	161,331	81,989	1.967	1.542	243,320
Emblica02	India	158,744	82,306	1.928	1.564	241,050
Emblica03	Bunga	137,584	73,494	1.872	1.365	211,078
Emblica04	Med Mayom	173,898	88,035	1.975	1.655	261,933
Emblica05	Phu Yai Sane	166,125	86,880	1.912	1.654	253,005
Emblica06	Pom Yak	171,237	87,133	1.965	1.437	258,370
Emblica07	To Lek	166,890	85,004	1.963	1.607	251,894
Emblica08	To Yak	161,850	82,638	1.958	1.69	244,488
Emblica09	Bang Berd	173,071	88,969	1.945	1.756	262,040
Emblica10	Mae Luk Dok	149,313	78,235	1.908	1.504	227,548
Emblica11	Pan Si Thong	137,829	71,801	1.919	1.364	209,630
Emblica12	Pan Siam	148,895	78,340	1.9	1.478	227,235
Emblica13	Pan Khao	115,097	59,672	1.928	1.111	174,769
Emblica14	the wild	129,554	66,605	1.945	1.27	196,159

**Table 3:** Extraction yields of crude and tannin-enriched extracts from five varieties of *Phyllanthus emblica*.

Accession	Weight of Extract (g)		Yield of Extract (% yield)	
	Crude Extract	Tannin-Enriched Extract	Crude Extract	Tannin-Enriched Extract
Emblica01	2.213	6.920	4.427	13.839
Emblica07	4.896	5.999	9.792	11.997
Emblica09	4.068	7.532	8.136	15.064
Emblica11	2.360	5.685	4.721	11.370
Emblica12	3.241	7.375	6.482	14.751

**Table 4:** Analysis of variance (ANOVA), phytochemical contents, enzyme inhibitory activities, and IC_50_ values of crude and tannin-enriched extracts from representative *Phyllanthus emblica* cultivars.

Extraction Method	Accession	Total Phenolic Content (mg GAE/g)	Total Tannin Content (mg/g)	α-Glucosidase Inhibition (%)	α-Glucosidase IC_50_ (mg/mL)	Pancreatic Lipase Inhibition (%)	Pancreatic Lipase IC_50_ (mg/mL)
Crude	Emblica01	15.530 ± 0.248 ^a^	333.095 ± 5.254 ^ab^	96.568 ± 0.421 ^b^	1.24 ± 0.08 ^c^	18.910 ± 0.846 ^b^	6.82 ± 0.31 ^bc^
	Emblica07	12.651 ± 0.208 ^b^	326.667 ± 5.303 ^bc^	97.989 ± 0.691 ^ab^	1.11 ± 0.05 ^c^	19.687 ± 0.832 ^b^	6.54 ± 0.27 ^bc^
	Emblica09	7.503 ± 0.101 ^d^	333.809 ± 4.410 ^ab^	96.501 ± 0.212 ^b^	1.29 ± 0.06 ^bc^	17.779 ± 1.833 ^b^	7.12 ± 0.42 ^b^
	Emblica11	9.989 ± 0.310 ^c^	323.809 ± 9.900 ^bc^	91.392 ± 1.083 ^c^	1.86 ± 0.12 ^a^	15.124 ± 1.958 ^b^	8.03 ± 0.58 ^a^
	Emblica12	6.583 ± 0.089 ^e^	312.619 ± 4.172 ^cd^	98.748 ± 0.251 ^a^	0.98 ± 0.04 ^d^	29.167 ± 1.069 ^a^	4.95 ± 0.24 ^d^
Tannin-enriched	Emblica01	9.031 ± 0.052 ^d^	316.429 ± 1.798 ^cd^	82.750 ± 0.869 ^a^	2.43 ± 0.15 ^a^	41.514 ± 1.310 ^ab^	2.72 ± 0.11 ^bc^
	Emblica07	10.482 ± 0.054 ^b^	292.857 ± 1.487 ^ef^	86.080 ± 2.819 ^a^	2.11 ± 0.14 ^ab^	38.776 ± 2.442 ^bc^	2.94 ± 0.13 ^b^
	Emblica09	8.147 ± 0.039 ^e^	301.429 ± 1.429 ^de^	58.865 ± 4.394 ^c^	4.38 ± 0.27 ^d^	33.403 ± 3.799 ^c^	3.46 ± 0.18 ^a^
	Emblica11	10.951 ± 0.053 ^a^	312.143 ± 1.487 ^cd^	71.257 ± 1.730 ^b^	3.08 ± 0.19 ^c^	20.001 ± 0.596 ^d^	5.94 ± 0.26 ^d^
	Emblica12	9.352 ± 0.141 ^c^	284.524 ± 4.232 ^f^	67.287 ± 2.114 ^b^	3.42 ± 0.21 ^bc^	46.263 ± 0.581 ^a^	2.11 ± 0.09 ^c^
	Capros^®^	81.713 ± 1.017	343.095 ± 4.212	87.943 ± 0.333	1.76 ± 0.09	1.973 ± 0.752	12.51 ± 0.84
	Significance	***	***	***	***	***	***
	Variety (V)	***	***	***	***	***	***
	Extraction (E)	***	***	***	***	***	***
	V × E	***	ns	***	**	**	**
	CV (%)	2.14	3.58	5.77	4.92	4.21	5.13

Data are presented as mean ± standard deviation (SD) of triplicate experiments. Different superscript letters within the same column indicate significant differences according to Duncan’s multiple range test (DMRT) at *p* < 0.05. ns = not significant; ** and *** indicate significance at *p* < 0.01 and *p* < 0.001, respectively. IC_50_ values were determined using dose–response analysis based on nonlinear regression of inhibition percentage against extract concentration.

**Table 5:** LC-MS/QTOF-based identification of phytochemical constituents in the ethanolic crude extract and tannin-enriched extract of *Phyllanthus emblica* (Emblica01).

No.	Analyte Peak Name/Library Hit	Retention Time	Relative Peak Area	Relative Abundance (%)
	The ethanolic crude extract			
1	L(+)-Arginine	1.23	4.188 × 10^5^	1.454
2	Proline	1.45	3.163 × 10^5^	1.098
3	Trigonelline	1.34	2.947 × 10^6^	10.235
4	Phosphorylcholine	1.29	2.072 × 10^5^	0.72
5	Vitamin C	1.39	5.935 × 10^5^	2.061
6	Pipecolic acid	1.66	9.203 × 10^6^	31.961
7	Pyrrolidonecarboxylic acid	1.66	1.353 × 10^6^	4.699
8	3-Phenylbutyric acid	1.84	1.966 × 10^5^	0.683
9	6-Hydroxypurine	1.98	1.106 × 10^5^	0.384
10	1-Methylhistidine	2.09	9.670 × 10^5^	3.358
11	Anisaldehyde	3.35	2.055 × 10^5^	0.714
12	Phenylalanine	3.01	1.088 × 10^6^	3.779
13	L-Tryptophan	5.65	2.875 × 10^5^	0.998
14	Adenine	5.96	2.387 × 10^5^	0.829
15	5-Methylthioadenosine	5.97	1.033 × 10^6^	3.588
16	(R,S) Epigoitrin	6.09	1.388 × 10^5^	0.482
17	Indoleacrylic acid	7.25	3.062 × 10^5^	1.063
18	2-Phenylbutyric acid	7.42	3.358 × 10^4^	0.117
19	Hamaudol Glycoside	8.62	2.391 × 10^5^	0.83
20	Syringaldehyde	10.11	1.464 × 10^5^	0.508
21	Myricetin	10.23	1.292 × 10^5^	0.449
22	Quercetin 7-rhamnoside; Vincetoxicoside B	10.73	2.628 × 10^5^	0.913
23	Cinnamic acid	11.19	1.453 × 10^6^	5.046
24	Quercetin-3′-O-glucoside	11.35	3.615 × 10^5^	1.255
25	Pulegone	11.44	2.212 × 10^5^	0.768
26	Quercetin	12.27	3.918 × 10^6^	13.607
27	Quercitrin	12.31	1.198 × 10^6^	4.161
28	Kaempferol	13.39	1.393 × 10^5^	0.484
29	Gingerglycolipid B +Na	13.47	3.299 × 10^5^	1.146
30	Pinocembrin	14.48	7.516 × 10^5^	2.61
	The tannin-enriched extract			
1	Proline	1.37	4.174 × 10^3^	0.104
2	Pipecolic acid	1.70	4.315 × 10^5^	10.764
3	3-Phenylbutyric acid	1.76	7.846 × 10^4^	1.957
4	Adenine	1.91	9.393 × 10^4^	2.343
5	6-Hydroxypurine	1.97	1.349 × 10^5^	3.365
6	Cantharidin	2.88	1.541 × 10^5^	3.844
7	Anisaldehyde	3.41	1.882 × 10^5^	4.695
8	Pantothenic acid	4.02	7.067 × 10^5^	17.629
9	Coumarin	4.68	6.624 × 10^4^	1.652
10	Indoleacrylic acid	5.66	3.112 × 10^5^	7.763
11	L-Tryptophan	5.66	8.538 × 10^4^	2.13
12	Camphor	5.45	1.421 × 10^4^	0.354
13	Pulegone	6.20	8.347 × 10^4^	2.082
14	Retinal	7.93	2.520 × 10^5^	6.286
15	Hyperin	9.34	2.516 × 10^5^	6.276
16	Ellagic acid	9.87	4.525 × 10^4^	1.129
17	2-Phenylbutyric acid	10.01	8.591 × 10^4^	2.143
18	3-N-butyl-4,5-dihydrophthalide	10.55	1.332 × 10^5^	3.323
19	Quercetin 7-rhamnoside; Vincetoxicoside B	10.83	2.070 × 10^5^	5.164
20	Amygdalin +NH_3_	11.47	1.403 × 10^5^	3.5
21	Gingerglycolipid B +Na	11.80	1.654 × 10^5^	4.126
22	Germacrene; (3E,7E)-3,7-dimethyl-10-propan-2- ylidenecyclodeca-3,7-dien-1-one	13.41	4.710 × 10^3^	0.117
23	Cinnamic acid	14.80	3.708 × 10^5^	9.25

Compounds were tentatively identified based on LC-MS/QTOF spectral library matching and retention time comparison. Relative peak area values represent the integrated ion intensity of detected metabolites, while relative abundance (%) was calculated as the percentage contribution of each metabolite peak area relative to the total detected peak area within each extract type. LC-MS/QTOF analysis was performed using three biological replicates of crude ethanolic and tannin-enriched extracts of *P. emblica* (Emblica01). Metabolite annotations remain putative and require further confirmation using authentic reference standards and MS/MS fragmentation analysis.

## Data Availability

The raw dataset in this study will be available, upon publication, in the Sequence Read Archive (SRA) repository with accession numbers SRR38265860, SRR38265848, SRR38265687, SRR38265686, SRR38265683, SRR38265682, SRR38265336, SRR38265335, SRR38265331, SRR38265328, SRR38264950, SRR38264913, SRR38264673, and SRR38264567.

## Supplementary Materials

The following supporting information can be downloaded at https://www.mdpi.com/article/10.3390/molecules31111786/s1, Table S1. The single-nucleotide polymorphism (SNP) genotyping profiles of all investigated *Phyllanthus emblica* accessions, providing a high-resolution view of genetic variation at the nucleotide level. Table S2. Pairwise patristic distances among 14 *Phyllanthus emblica* accessions inferred from the Neighbor-Joining (NJ) phylogenetic analysis based on GBS-derived SNP sequences. Table S3. Pairwise patristic distances among 14 *Phyllanthus emblica* accessions inferred from the unweighted pair group method with arithmetic mean (UPGMA) analysis based on GBS-derived SNP sequences. Table S4. Representative TICs, EICs, and MS/MS spectral information of major metabolites identified in crude ethanolic extract (CEO) and tannin-enriched extract (TEO) of *Phyllanthus emblica* (Emblica01).
